# Methods of Identifying *Gordonia* Strains in Clinical Samples

**DOI:** 10.3390/pathogens11121496

**Published:** 2022-12-08

**Authors:** Ekaterina Frantsuzova, Alexander Bogun, Anna Vetrova, Yanina Delegan

**Affiliations:** 1 Institute of Biochemistry and Physiology of Microorganisms, Federal Research Center “Pushchino Scientific Center for Biological Research of Russian Academy of Sciences” (FRC PSCBR RAS), 142290 Pushchino, Moscow Region, Russia; 2 State Research Center for Applied Microbiology and Biotechnology, 142279 Obolensk, Moscow Region, Russia

**Keywords:** *Gordonia*, pathogenesis, NGS, identification, sequencing, phylogenetic markers, MALDI–TOF MS

## Abstract

*Gordonia* spp. are members of the family *Gordoniacea* in the suborder *Corynebacteriales*; their habitat, in most cases, is soil. Many representatives of this genus are human or veterinary pathogens. The main cause of the lack of a standardized approach to dealing with infections caused by *Gordonia* is their erroneous identification and little information regarding their susceptibility to antimicrobial drugs. This review presents the most common methods for identifying *Gordonia* strains, including modern approaches for identifying a species. The main prospects and future directions of this field of knowledge are briefly presented.

## 1. Introduction

*Gordonia* spp. are members of the phylum *Actinomycetota*, class *Actinobacteria*, order *Actinomycetales*, suborder *Corynebacterineae*, and the family *Gordoniacea* [1]. Their habitat, in most cases, is soil. Modern literature concerning *Gordonia* can be divided into several areas, the main of which is the study of the ability of these organisms to utilize various pollutants. *Gordonia* strains capable of utilizing alkanes [2,3,4], polycyclic aromatic compounds (PAHs) [5,6,7], thiophenes [8,9,10,11], and rubber [12,13,14] are known. The metabolic flexibility of *Gordonia* strains puts them on par with *Rhodococcus*, known degraders of various pollutants.

According to the List of Prokaryotic Names with Standing in Nomenclature (https://lpsn.dsmz.de/ (accessed on 30 October 2022)), the *Gordonia* genus currently consists of 53 species, four of which (“*Gordonia australis*”, “*Gordonia jacobaea*”, “*Gordonia pseudoamarae*”, “*Gordonia terrea*”) are not validated, and *Gordonia nitida* is a later heterotypic synonym of *Gordonia alkanivorans* [15]. In addition to the ability to degrade organic pollutants, *Gordonia* strains are interesting as producers of secondary metabolites, biosurfactants (glycolipids [16,17], lipopeptides [18], and lipoglycoproteins [19,20]), and carotenoids [21,22,23,24].

Although most *Gordonia* species have been isolated from environmental samples, several members of this genus were initially isolated from clinical specimens and are considered opportunistic human pathogens [25] (Table 1).

Other cases of *Gordonia* pathogenesis have been detected in species, representatives of which are usual environmental strains capable of degrading various pollutants (Table 2).

Thus, representatives of 18 of the 53 *Gordonia* species may be pathogenic to humans and animals.

In most cited sources, patients were immunosuppressed, and infections by *Gordonia* species occurred secondarily [25]. However, there are also cases of people not immunocompromised and having no medical device but being affected by *Gordonia* [49,50]. Cases of *Gordonia* lesions are most common in adults, but several examples of infections in children are known. For example, in 1994, a 3-year-old boy presented with a brain abscess caused by *G. terrae* [51]. Blaschke et al. [52] observed inflammatory processes in children; in all cases, the cause was *Gordonia* strains, e.g., *G. terrae*, *G. otitidis*, and *G. bronchialis*. In the work of Ramanan et al. [53], one of the cases of infection was also in a child; the strain was identified as *Gordonia* sp.

The *Gordonia* strains themselves are not the cause of death. However, in most cases, pathogenic strains infect immunocompromised patients, and death most often occurs from a general worsening of the state of health [54,55].

There are also cases when, as a result of antibiotic therapy, the symptoms of the *Gordonia* lesion disappeared, but after a while, the patient died from progressive disease [40,56,57,58].

Bloodstream infections caused by *Gordonia* strains are often detected in patients with medical devices (Hickman central venous catheter (CVC), etc.) [54,59]. Extracellular biosurfactants with a polysaccharide structure help them form biofilms [60]. This allows *Gordonia* strains to adhere to rubber material in catheters [61]. Werno et al. [62] revealed that biofilms also reduce the penetration of antibiotics and contribute to the emergence of drug resistance in *Gordonia* strains.

*Gordonia* strains can cause infections in other animals besides humans. In veterinary medicine, they are known as the cause of mastitis, mainly in cows [38].

The present review aims to summarize the main modern methods of identifying *Gordonia* strains and provides a brief summary of therapy approaches.

## 2. Biochemical Approaches

The representatives of the *Gordonia* genus are slow-growing, and biochemical identification of these strains is time-consuming, labor-intensive, and usually inconclusive [63]. The biochemical parameters for the discrimination of *Gordonia* strains from other actinomycetes include the following: acid-fast staining, evaluation of aerial hyphae and the sensitivity to lysozyme, the production of nitrate reductase, and urea hydrolysis [1,64].

### 2.1. Medium Engineering

For *Gordonia* cultivation, standard microbiological media, including solid Löwenstein–Jensen, Columbia agar with 5% sheep erythrocytes, Sabouraud agar, chocolate agar, and liquid Schaedler medium, are often used [65,66,67]. Cultivation is usually performed on agarized media, as this allows the evaluation of the appearance and purity of the isolates.

*Gordonia* strains are slow-growing. Franczuk et al. [66] observed the growth of strains on Columbia blood agar after at least 48 h and on Löwenstein–Jensen medium after 4 days of growth. In the work of Jannat-Khah et al. [67], strains began to grow on day 3 of incubation, and active growth was observed on day 11. Iida et al. [26] cultivated *Gordonia* strains on Muller–Hinton agar slants for 1 week. The slow growth of *Gordonia* strains is a significant problem since it takes a long time to determine a correct diagnosis and begin therapy.

The strains, in most cases, have a characteristic appearance [68]. Franczuk et al. [66] observed creamy-yellowish colonies on Columbia agar, and the growth of yellow–orange colonies was noted on Löwenstein–Jensen solid medium. However, the appearance alone is insufficient to identify *Gordonia* strains because some *Rhodococcus* species may look the same.

### 2.2. Biochemical Test

To determine enzymatic activities, API CORYNE strips are used; however, using this method, the *Gordonia* strains are usually identified as *Rhodococcus* [57,69] or *Nocardia* and other actinomycetes [65].

The API CORYNE (BIOMERIEUX) system is a set of 20 micro-tubes that contain dehydrated substrates. The kit is designed to demonstrate (a) enzymatic activity and (b) the fermentation of carbohydrates. Strips for enzymatic reactions are inoculated with a concentrated suspension of microorganisms. During incubation, metabolic reactions in bacteria provoke a color change. Strips for fermentation, in turn, are inoculated with an enriched medium with a pH indicator. Fermentation reactions provoke acidification of the medium, as a result of which the color of the pH indicator changes.

In addition to enzymatic reactions, substrate utilization profiling is sometimes used. This method, along with the API CORYNE enzymatic assay, can be attributed to biological fingerprinting approaches. For substrate utilization profiling, either cultivation with the substrate is used [70] or test systems, for example, the Biolog GP2 MicroPlate assay [26]. This system is based on redox chemistry. This chemistry reacts to bacterial respiration, not the appearance of metabolites, and therefore does not need the addition of colored reagents.

## 3. Matrix-Assisted Laser Desorption Ionization–Time of Flight (MALDI–TOF) Mass Spectrometry (MS)

Matrix-assisted laser desorption ionization–time of flight (MALDI–TOF) mass spectrometry (MS) is an automated method recently implemented as a tool for bacterial identification in clinical microbiology laboratories [71]. This method of identifying *Gordonia* strains in clinical samples began to be used earlier than sequencing and currently remains in demand. Among the advantages of MALDI as a method of bacterial identification, Alcolea-Medina et al. [72] point out that it is simpler and cheaper than whole-genome sequencing (WGS).

According to the standard Bruker score system, for the identification of species, the score value must be ≥2.0, for the genus, between 2.0 and ≥1.7, and if there is a score of ≤1.7, identification is considered unreliable [73]. Due to the poor identification of *Gordonia* strains in accordance with generally accepted score values, some authors suggest lowering the cutoff thresholds so that they are ≥1.5 and ≥1.7 for the genus and species, respectively. Thus, Rodriguez-Lozano et al. [74] identified *G. bronchialis* with a score value of 1.72 (Bruker Biotyper ver. 3.1), which was lower than that defined in the manufacturer’s criteria (>2.00) for the acceptance of identification at the species level. The MALDI identification results were validated using 16S rRNA sequencing.

Similar results were previously obtained by Barberis et al. [75] when analyzing a collection of Gram-positive rods of clinical origin, including three *Gordonia* strains. Only one *G. terrae* strain was identified with a score >2.0 (Biotyper ver. 3.1). Ercibengoa Arana et al. [71] demonstrated that when the cutoff threshold is reduced to ≥1.5 and ≥1.7 for genus and species, respectively, the number of cases of successful identification of *Gordonia* strains increases significantly, from 10 to 20 isolates at the genus level and from 2 to 11 isolates at species level. In ref. [71], measurements were taken in three repetitions, and the average of the three results was used for further analysis.

Researchers believe that the main reason for the erroneous identification of *Gordonia* strains when using the MALDI method is the lack of information regarding many species in the Bruker database (Table 3).

Thus, in the work of Hsueh et al. [81], three *Gordonia amicalis* were mistakenly identified as *G. rubripertincta* because *G. amicalis* is not included in the Bruker Biotyper 3.1 software (database [DB] 5627). Ercibengoa Arana et al. [71] misidentified four *G. otitidis* isolates, probably because of the absence of any *G. otitidis* reference isolate in the Bruker database. Ercibengoa Arana et al. [71] used the following reference strains available in the Bruker database: *Gordonia aichiensis*, *G. alkanivorans*, *G. australis*, *G. bronchialis* (n = 2), *G. rubripertincta* (n = 11), and *G. sputi* (n = 4). The isolates studied by Lam et al. [79] and Ding et al. [40] were identified as *Gordonia* sp. Identification of the species was not performed because the strains did not correspond sufficiently to any species in the Bruker database (Bruker Biotyper ver. 3.1). Bzdil et al. [38] point out that MALDI Biotyper MBT 4.1 currently contains six *Gordonia* spp. only (*G. aichiensis*, *G. alkanivorans*, *G. bronchialis*, *G. rubripertincta*, *G. sputi,* and *G. terrae*).

## 4. Chromatographic Approaches

### 4.1. High-Performance Liquid Chromatography (HPLC)

The genera *Gordonia*, *Nocardia*, and *Rhodococcus*, belong to the cell wall chemotype IV. Their whole-cell sugar pattern is characterized by the presence of meso-diaminopimelic acid (meso-DAP), arabinose, and galactose in cell hydrolysates [82]. The genera of this cell wall type are classified based on the presence or absence of mycolic acids and the molecular weights of the mycolates [83,84].

Analysis of the profile of mycolic acids of *Gordonia* strains is sometimes used to identify them to a genus and is useful for separating *Gordonia* from the genera of other *Actinobacteria* [54]. However, previously [51], it was revealed that the mycolic acid pattern of the *Gordonia* strain, as assayed by HPLC, was similar to that of the *Nocardia* representatives. The mycolic acids of *Nocardia* species have 46–58 carbon atoms [83,85].

According to the profile of mycolic acids, it is possible to distinguish *Gordonia* from *Rhodococcus*. *Gordonia* strains have mycolic acids with 44–66 carbon atoms and dehydrogenated menaquinones with 9 isoprene units. The mycolic acids of *Rhodococcus* strains, on the other hand, have 34–52 carbon atoms and dehydrogenated menaquinones with 8 isoprene units [60]. The HPLC method is not suitable for the fast and accurate identification of *Gordonia* strains in clinical samples, especially to the species, and therefore is currently rarely used for this purpose and only together with genetic approaches.

### 4.2. Thin-Layer Chromatography

The thin-layer chromatography method for identifying aerobic actinomycetes was first used a long time ago [86,87]. The method consists of separating diaminopimelic acid (DAP) and sugars isolated from cells on a chromatographic plate, followed by staining of these compounds [88,89]. DAP visualization is performed by spraying a sample with 0.2% ninhydrin in acetone and heating at 100 °C for 3 min; sugar visualization is conducted by spraying the chromatogram with acid aniline phthalate and heating at 100 °C for 4 min.

TLC for identifying *Gordonia* by DAP and sugar was used by Aoyama et al. [90] along with 16S rRNA gene sequencing. All 31 strains were determined by TLC pattern analysis to contain N-type mycolic acid, which is characteristic of *Gordonia*, *Nocardia*, and *Rhodococcus*. All strains contained arabinose and galactose as characteristic components in addition to meso-DAP [91].

Hamid et al. [44] identified a *Gordonia* strain isolated from a Zebu cow with lymphadenitis. Among the approaches used were TLC, 16S rRNA gene sequencing, and DNA–DNA hybridization. On thin-layer chromatography, the organism contained mycolic acids that co-chromatographed with mycolates from *Gordonia* species. The strain was determined as *G. sinesedis* with the help of genetic methods.

TLC is not a method accurate enough to identify strains to species based on its results. In addition, it does not meet modern requirements for the rapidity of analysis, and labor costs are inferior to other methods, particularly genetic ones. In this regard, TLC is now rarely used in clinical practice, as well as HPLC.

## 5. Genetic Approaches

### 5.1. Amplification and Sequencing of Phylogenetic Markers

Despite the spread of genetic methods for identifying microorganisms in various fields of biological science and industries, these methods have been used in medicine relatively recently. Identifying *Gordonia* strains in clinical samples by sequencing the main phylogenetic markers and analyzing the results has only become widely used in the last 10–15 years [52,54,69].

As phylogenetic markers for identifying microorganisms, housekeeping genes are usually used. Housekeeping genes are necessary to maintain the most important vital functions of the organism. The housekeeping genes most commonly used as phylogenetic markers are as follows: 16S rRNA, *gyr*B, *rpo*B, *cat*, and *ppk* [92]. The sequencing and analysis of additional phylogenetic markers, i.e., more specific genes that are not housekeeping, is a more specific task and mainly serves to distinguish closely related species within species groups [93]. Specific phylogenetic markers are not used to identify *Gordonia* in clinical samples.

#### 5.1.1. 16S rRNA

The most commonly used phylogenetic marker for identifying *Gordonia* is the 16S rRNA gene because it contains both highly conserved and hypervariable regions [94]. In addition, the advantage of using this gene as a marker is that reference databases have been compiled for analysis [95,96,97,98].

To identify the *Gordonia* strains, both an almost complete gene and only variable regions are sequenced. In the work of Franczuk et al. [66], which aimed to identify *G. bronchialis,* the V3-V4 region of the 16S rRNA gene was amplified with the use of primer “forward” 5′-ACTCCTACGGGAGGCAGCAG-3′ and primer “reverse” 5′-TACCAGGGTATCTAATCC-3′. Wang et al. [99] performed rapid metagenomic next-generation sequencing of 16S rRNA to determine *G. aichiensis* in a patient with peritoneal dialysis-associated peritonitis.

However, in most cases, sequencing the longest possible fragment of the 16S rRNA gene of individual isolates is performed (Table 4).

At the same time, 16S rRNA amplicon sequencing has several disadvantages:

1. Since the 16S rRNA gene is very conservative and evolves extremely slowly, it cannot be used to separate closely related strains and identify them.
2. During the amplification of 16S rRNA, the formation of chimeric amplicons is possible [109,110].
3. The number of copies of the 16S rRNA gene in different species is variable, and single-nucleotide polymorphisms (SNPs) at the single-cell level may result in an overestimation of diversity [111].
4. In the case of closely related species, it is difficult to delineate species in cluster analysis [112].

Due to the above-mentioned features of the use of the 16S rRNA gene for identifying microorganisms, in some cases, it is necessary to use additional phylogenetic markers. In the case of *Gordonia* strains, the *gyr*B, *sec*A, and *hsp*65 genes are most often used [50,79,105].

#### 5.1.2. *gyr*B (gyrase B)

The threshold for assigning bacteria to a species based on the *gyr*B sequence is lower than 16S rRNA. This is due to the greater variability of the *gyr*B gene relative to 16S rRNA. Currently, for *gyr*B phylogeny, the species threshold is not clearly marked; it is different for different groups of organisms [113]. According to Kang et al. [114], the species threshold for *Gordonia* is 77.5–97.6%.

DNA gyrase plays an important role in DNA replication [115]. Both A and B subunits of DNA gyrase can be used as phylogenetic markers. Thus, *gyr*A is often used to identify bacilli [116,117]. However, we have not found any examples of the use of *gyr*A as a marker for the identification of *Gordonia* strains in the literature. Perhaps the preferred use of *gyr*B for this purpose is due to a more meticulous methodological base (primers and amplification modes) and a large number of reference sequences of *Gordonia gyr*B with which the data obtained can be compared in databases.

Shen et al. [118] identified *Gordonia* strains using *gyr*B and 16S rRNA gene sequences. A pair of primers for the amplification of the *gyr*B gene was developed using conserved amino acid sequences MTQLHAG and KLRYHKIV of *E. coli* DNA gyrase (Table 5). The GYRB1 sequence has a single amino acid replacement in comparison with the primer UP-1 from the UP-1/UP-2 pair [119,120], which are the most commonly used classical primers for the amplification of *gyr*B. The reverse primer was newly designed.

In the work of Jannat-Khah [67], the *Gordonia araii* strain was identified by *gyr*B sequencing. This is the first case of detecting a pathogenic strain of *G. araii* causing infection associated with a medical device. For *gyr*B gene amplification, the primer pair GYRB1/GYRB2 [118] was used.

Johnson et al. [121], using sequencing and analysis of 16S rRNA and *gyr*B genes, identified *G. bronchialis* in isolates obtained from a 52-year-old woman with a history of Hodgkin’s lymphoma, prior splenectomy, and breast cancer. For the amplification of the *gyr*B gene, the primer pair GYRB1/GYRB2 [118] was used. Both patient isolates were 100% similar and 93% related to the type strain of *G. bronchialis*. Similarly, *G. bronchialis* was identified in the work of Akrami et al. [65] in a 69-year-old man with erythema and drainage at the cranial end of the sternotomy incision. PCR amplification of the DNA gyrase region of *Gordonia* spp. was carried out with a set of novel PCR primers; however, the primer sequences were not specified.

The strains of *Gordonia* species that rarely cause human infections are also known to be identified using *gyr*B. So, Ding et al. [40] identified *Gordonia polyisoprenivorans* as a cause of catheter-related bacteremia in an AIDS patient. Sequencing of the *gyr*B genes was performed according to a previous report [6] using the GYRB1/GYRB2 primer pair.

Negishi et al. [105] found *G. sputi* in a sample from an 18-year-old man with acute lymphocytic leukemia. Here, identification was also performed using two markers, 16S rRNA and *gyr*B; however, here, unlike the works mentioned above with *gyr*B, amplification was performed not with GYRB1/GYRB2 primers but according to the work of Kang et al. [114]. Authors revealed that *G. sputi* could not be distinguished from *G. aichiensis* or *G. otitidis* using 16S rRNA gene sequence analysis alone but could be successfully discriminated against using the *gyr*B gene sequence.

Kang et al. [114], for *gyr*B amplification, used both primers UP [120] and the F1/R1296 pair, as well as the *gyr*B sequence of *Nocardia farcinica* IFM 10152 [122]. The primer F1 is a modified and more specific version of UP1. Later, the F1/R1296 pair was used for the amplification of *gyr*B of *Nocardia* [123]. Modified primers UP are also known [50]; they were used to identify *G. otitidis* in the sputum sample taken from a 76-year-old woman suspected of having nontuberculous mycobacterial (NTM) lung disease. For the sequencing of the amplicon primers, sF1 (5′-GAG GTC GTC ATG ACC CAG CTG CA-3′) and sR1296-modi (5′-AGC AGC GTC GAG ATG TGC TGG CC-3′) were used.

Among the disadvantages of using *gyr*B as a phylogenetic marker, Kang et al. [114] mention that gyrases of not all *Gordonia* species can be amplified using a pair of F1/R1296 primers. A similar drawback was also noted in the UP pair [118]. Currently, the most commonly used pair of primers for identifying *Gordonia* in clinical samples is GYRB1/GYRB2.

Another disadvantage of using *gyr*B as a phylogenetic marker is establishing similarity cutoff criteria for reliable and accurate identification since intraspecies sequence variability has been observed [124]. Authors cite the heterogeneous species *Gordonia terrae*, where the similarity of different strains with type one can vary at 87–98%. Authors suggest that in the case of heterogeneous species, other (non-*gyr*B) phylogenetic markers and DNA–DNA hybridization may be useful for identification.

#### 5.1.3. *sec*A, *hsp*65

In some cases, *sec*A, not *gyr*B, is used to identify clinical samples of *Gordonia* in addition to 16S rRNA. SecA is an ATPase that plays an essential role in the protein translocation mechanism [125,126]. Both *gyr*B and *sec*A provide a greater discrimination degree compared to 16S rRNA. According to Lasker et al. [124], interspecies sequence similarity for the *sec*A gene sequence and the SecA amino acid sequence was 81.9–98.0% and 83.0–100.0%, respectively. The amino acid motif constructed based on the *sec*A1 amino acid sequences provides the opportunity to discriminate the genus *Gordonia* from related pathogenic actinomycetes such as *Nocardia*, *Streptomyces*, and *Mycobacterium* [114].

Lam et al. [79] used *sec*A as an additional (in addition to 16S rRNA) marker for identifying *Gordonia* strains in samples taken from four patients with continuous ambulatory peritoneal dialysis-related peritonitis. For amplification, a pair of SecA1-f/SecA1-r (Table 6) primers [114] was used. These primers were developed using data regarding the gene sequences of *Mycobacterium* and *Nocardia* in GenBank [127,128]. The sequencing and analysis of 16S rRNA and *sec*A amplicons successfully identified *G. sputi* in two patients and *G. bronchialis* in one patient. As for the isolate detected in the fourth patient, its 16S rRNA gene had 99.7% identity to that of the *Gordonia terrae* type strain, but the *sec*A1 gene had 94.0% sequence identity to that of the *Gordonia lacunae* type strain. The authors suggested that it was a novel *Gordonia* species [79].

Given the difficulties in identifying individual *Gordonia* isolates even when using two markers, several researchers propose a multilocus analysis of at least three markers. The most commonly used combination is 16S rRNA, *gyr*B, and *sec*A. For the first time, this approach was used by Kang et al. [114]. The authors noted that sometimes there was no signal during the amplification of *gyr*B in some species using primers that were successfully used for other species. The length of the *gyr*B amplicons during amplification with the same primers turned out to be different: in most *Gordonia,* it was 1230 bp, and in *G. otitidis*, 1188 bp.

Using the third marker (*sec*A) solved the problem with *gyr*B. Universal primers have been developed to amplify the 496 bp region of the *sec*A1 gene (Table 4).

Kim et al. [50], to identify nontuberculous mycobacteria (NTM) in the sputum sample taken from a woman with lung disease, used genotypic identification targeting an internal transcribed spacer (ITS). The strain was determined by this method as *Mycobacterium lenti-flavum* or *Mycobacterium genavense*. However, sequencing of 16S rRNA, gyrB, and secA1 genes revealed that the strain belonged to *G. otitidis*. In this regard, it can be concluded that the method convenient for mycobacteria is not suitable for *Gordonia*.

There are known examples of identifying *Gordonia* by analyzing the 441 bp fragment (Telenti segment) of the *hsp*65 gene [129]. This method was first proposed by Telenti et al. [130] and is widely used for identifying mycobacteria [131,132], but it is used infrequently for *Gordonia*. Unlike the phylogenetic markers described above, for which the main stages of work consisted of amplification and sequencing, in the case of *hsp*65, restriction analysis is assumed.

Lai et al. [103] analyzed the strains of *Gordonia*, which were the causative agents of infections, at a medical center in Taiwan from 1997 to 2008. To identify the strains, the authors used 16S rRNA gene sequence analysis and restriction profiles of the 440 bp fragment of the 65 kDa heat shock protein gene (*hsp65*) amplified using the primers TB11 (5′-ACCAACGATGGTGTGTCCAT-3′) and TB12 (5′-CTTGTCGAACCGCATACCCT-3′) [129,133]. Hydrolysis of amplicons was performed with the help of enzymes M*sp*I and *Hin*fI. Using this method, *G. terrae* and *G. sputi* were identified. Chang et al. [77] and Wright et al. [101], using 16S rRNA and *hsp*65, identified *G. bronchialis* in a case of deep sternal wound infection with sternal osteomyelitis after open-heart surgery.

In addition to housekeeping gene analysis (16S rRNA, *hsp*65), Lai et al. [103] used random amplified polymorphic DNA (RAPD) analysis performed using primers M13 (5′-TTATGTAAAACGACGGCCAGT-3′) and H4 (5′-GGAAGTCGCC-3′). The isolates from different patients had different patterns, indicating that they were genetically unrelated, and that nosocomial transmission had not occurred. In this paper, RAPD analysis was used for the first time to study the clinical isolates of *Gordonia*.

### 5.2. DNA–DNA Hybridization

Among the genetic methods not related to sequencing, DNA–DNA hybridization can be noted. The use of hybridization for species identification is rational when the number of candidate species is reduced by other methods to 1–2; otherwise, the approach becomes methodical and time-consuming. Verma et al. [54] identified *G. polyisoprenivorans* in a blood sample of a 78-year-old male with a medical history of multiple episodes of gastrointestinal bleeding secondary to Osler–Weber–Rendu and myelodysplastic syndrome with pancytopenia. The authors used 16S rRNA gene sequencing and DNA–DNA hybridization. *G. polyisoprenivorans* are infrequent human pathogens. Usually, these are soil strains that, due to their ability to recycle rubber, have the potential for use in biotechnology [134]. However, it is known that *G. polyisoprenivorans* strains can form biofilms on the catheter surface [59,135,136].

DNA–DNA hybridization was used by Jannat-Khah et al. [67] to confirm the results obtained by sequencing the 16S rRNA and *gyr*B genes. The high degree of sequence similarity (99.6%) between the 16S rRNA gene sequences for isolate W8543 and the *G. araii* type strain allowed them to identify the isolate as *G. araii*, and DNA–DNA hybridization analysis supported the results.

### 5.3. Next-Generation Sequencing (NGS) for Gordonia Identification

The *Gordonia* strains continue to be considered rare pathogens, but the problem of identifying these strains in clinical samples remains unresolved. Multilocus sequencing of amplicons currently remains the most accurate of the methods used for the taxonomic determination of strains; however, it takes time, and, in addition, there is always a possibility that the selected amplification mode will not work for a particular sample.

Sequencing whole genomes is a promising method for the rapid and accurate identification of *Gordonia* strains. At the same time, replenishing databases with genomes of *Gordonia*, including genomes belonging to clinical strains, will make it possible to operate with more material for genome comparison in the future.

When identifying strains based on the whole genome sequence, modern in silico methods, such as digital hybridization (DDH), can be used [137]. If the genomic DNA of two organisms reveals a DDH similarity of below 70%, these strains are considered representatives of distinct species and vice versa [138,139].

There are few examples of sequencing genomes of pathogenic *Gordonia* in modern literature. Koenigsaecker et al. [140] sequenced the strain *Gordonia* sp. UCD-TK1 isolated from a patient chair in a surgery center (Redding, CA, USA). The genome was sequenced on Illumina MiSeq (2 × 300) and assembled using an A5 assembler [141] to 95 scaffolds with 22× coverage. The greatest similarity in the 16S rRNA gene was observed with representatives of *G. terrae* and *G. lacunae*. However, the authors did not carry out the definition of the species due to the lack of data on the complete genomes of other representatives of *G. lacunae*, which could be used as a reference.

Gulvik et al. [142] sequenced the *Gordonia* sp. strain X0973 obtained from a human abscess. The authors used Illumina MiSeq and PacBio reads to obtain complete assembly. As a result, a complete circular genome sequence of 3.75 Mbp was obtained. Initially, the strain was identified as *Gordonia* by 16S rRNA gene sequencing and previously assigned to the species *G. amarae*. It is further stated that the average nucleotide identity (ANI) of the isolate with *Gordonia* type strains is in the range of 80.9 ± 5.4%, and DDH with *Gordonia araii* type strain (which is considered as the closest relative) is 22.6%. Such a conclusion is somewhat unusual, given that the species threshold for DDH is at least 70% [137].

Bzdil et al. [38] studied a variety of *Gordonia* strains isolated from the milk of dairy cows with mastitis. Nine isolates were identified as *G. paraffinivorans* by whole-genome sequencing. An interesting fact is that there are no examples of the pathogenic effects of *G. paraffinivorans* on humans in the literature; however, in veterinary medicine, there are several other reported infections caused by this genus. *G. paraffinivorans*, similar to *G. polyisoprenivorans*, is primarily interesting as a promising biotechnological agent for the degradation of hydrocarbon pollutants and is rarely the cause of diseases. Ahrholdt [143] revealed *G. paraffinivorans* as a cause of mastitis in cows in Saxony, Germany. Similar work was performed by Valkó [144] in Hungary, where the representatives of *G. paraffinivorans* caused the same disease in cows. In both cases, pathogens were identified by the sequencing and analysis of 16S rRNA genes.

In all nine samples, the authors [38] identified genes encoding virulence factors similar to those of *Mycobacterium tuberculosis* and *Mycobacterium smegmatis*. However, the authors noted that five genes similar to the virulence genes of *Mycobacterium* were found in the genome of the isolates from mastitis cases and both environmental reference strains. From the results obtained, it can be assumed that the pathogenicity or non-pathogenicity of strains is not so much due to the presence of certain genes as the expression of these genes. This assumption may be supported by the fact that there are not only pathogenic or non-pathogenic species among *Gordonia*; in each case, we are talking about specific representatives.

## 6. Treatment of Diseases Caused by *Gordonia* Strains

Infections caused by *Gordonia* spp. are often difficult to diagnose [49]. This is due to erroneous identification using a phenotypic test and slow growth of strains (approximately 3 days). The biochemical analysis used to determine the species is often unreliable [1]. However, most infections are resolved after the removal of the infected medical device and antibiotic treatment, resulting in the successful recovery of the patients [25]. The main advantage of this approach is that *Gordonia*, unlike related actinobacteria, does not have multiple antibiotic resistance [38,52]. The disadvantage is that there is no single treatment regimen for infections of *Gordonia* strains, and for each case, treatment must be selected.

Sometimes antibiotic therapy is prescribed to the patient empirically, according to symptoms, without waiting to identify the pathogen organism and determine the spectrum of antibiotic resistance. For example, in the work of Brust et al. [63], one of the patients was initially started on vancomycin and piperacillin/tazobactam, but this was changed to imipenem, amikacin, and minocycline, and the results of determining the strain as *G. sputi* were obtained when the therapy had already been started. A similar approach was used by Ramanan et al. [53] and Negishi et al. [105].

There are cases when, due to the erroneous definition of *Gordonia* as *Nocardia*, *Mycobacterium*, etc., the treatment was ineffective. Thus, trimethoprim–sulfamethoxazole is often used to treat *Nocardia* infections [145], and Blaschke et al. [52] show that it is ineffective against *Gordonia*.

Jeong Rae et al. [55] mistakenly identified the pathogen strain by biochemical methods as *Corynebacterium*, and treatment was selected in accordance with the approaches adopted for *Corynebacterium* strains: the authors used vancomycin, ceftriaxone, and clindamycin combination, and piperacillin/tazobactam, but the patient’s condition did not improve, and only after that the authors used 16S rRNA gene sequencing to identify the strain as *Gordonia*.

Martin et al. [78] suspected *Listeria monocytogenes* infection in a patient (by biochemical and microbiological examination of cerebrospinal fluid). The treatment was selected based on experience with *Listeria*: ampicillin, associated with vancomycin and ceftriaxone. However, the patient persisted with fever, and therapy was changed to linezolid and meropenem to cover the possibility of nosocomial secondary infection [78]. After, the pathogen strain was identified correctly (*Gordonia sputi*) using the MALDI TOF MS method.

The correct identification of the pathogenic organism to the species will allow, first, not to waste time on therapy designed for other groups of organisms and ineffective for *Gordonia*. Second, it will make it possible to use the experience of predecessors to successfully choose antibiotics for various species of *Gordonia*. Parallel to the identification of the microorganism, clinicians usually carry out antibiotic resistance testing. Most of the known *Gordonia* strains are susceptible to amoxicillin-clavulanate, amikacin, ceftriaxone, ciprofloxacin, linezolid, meropenem, minocycline, trimethoprim-sulfamethoxazole, tigecycline, and vancomycin [57,67,121]. At the same time, Moser et al. [136] revealed that the *G. polyisoprenivorans* strain had poor susceptibility to trimethoprim-sulfamethoxazole, clarithromycin, tigecycline, and minocycline. In ref. [65], the strain of *G. bronchialis* was susceptible to penicillin, gentamicin, levofloxacin, minocycline, vancomycin, linezolid, tetracycline, and erythromycin. It can therefore be concluded that despite the lack of a universal approach to the treatment of *Gordonia* infections, there is still some information regarding the use of antibiotics against different *Gordonia* species.

It is known that *G. otitidis* can be successfully treated with meropenem and ceftriaxone [68], imipenem, and clarithromycin [52]. Vancomycin is used against *G. terrae* [146], sometimes in combination with ceftazidime [147] or rifampin [52], as well as ciprofloxacin [52]. Treatment of *G. polyisoprenivorans* infections is often achieved using imipenem, ciprofloxacin, and vancomycin [40,54]. We believe that selecting effective antibiotics without the need for a “trial-and-error” approach will avoid the appearance of multidrug resistance in *Gordonia* in the future.

## 7. Prospects for Working with Infections Caused by *Gordonia* Strains

Recent studies show that the problem of identifying *Gordonia* strains in clinical samples remains unresolved. Based on the works reviewed, the following trends in this field can be formulated:

1. Chromatographic methods and mass spectrometry are currently rarely used to identify *Gordonia* strains. Chromatographic methods fail to distinguish them from related genera (*Nocardia* and *Rhodococcus*).
2. Currently, MALDI–TOF MS is the main instrument for identifying *Gordonia* in clinical samples. The accuracy of this method in detecting the genus is proven, but MALDI–TOF MS is unsuitable for accurately identifying *Gordonia* as a species.
3. The analysis for antibiotic resistance is necessary to accumulate information regarding the resistance/sensitivity of different *Gordonia* species to antibiotics. Depending on this information, the treatment of infections caused by each species of *Gordonia* is selected.
4. At the moment, the most accurate identification result can be obtained either by multilocus sequencing of housekeeping genes (16S rRNA, *gyr*B, and *sec*A) or by whole-genome sequencing. There is reason to believe that these methods will become increasingly widespread.

The correct determination of the taxonomic position of strains allows, on the one hand, to quickly select effective therapy based on the experience of predecessors and, on the other, to replenish the pool of methodological approaches to treat *Gordonia* infections of different species.

## 8. Conclusions

*Gordonia* spp. are common environmental bacteria with flexible genome structures and diverse metabolic capabilities. Despite their attractiveness for use in various fields of biotechnology, researchers are increasingly concerned about the growing number of clinical cases of infection with *Gordonia* strains, especially in immunocompromised patients. The main problems of working with *Gordonia* in medicine today are the complexity of their rapid and accurate identification and the lack of a universal therapy regimen.

The cases analyzed reveal the importance of molecular analysis in the definitive identification of these and other related species. The spread of genetic methods of identification of *Gordonia* strains suggests that in the near future, it will be possible to solve the problem of the erroneous identification of these strains.

## Figures and Tables

**Table 1:** Information concerning *Gordonia* species whose type strains were newly isolated from clinical samples.

Species	Type of Sample	Disease	Identification Approach	Reference
*Gordonia otitidis*	ear discharge	external otitis	mycolic acid profile, compound utilization patterns, 16S rRNA gene sequencing	[26]
*Gordonia araii*	sputum	bacterial pneumonia	TLC, HPLC, 16S rRNA gene sequencing, DNA–DNA hybridization	[27]
*Gordonia effusa*	sputum	kidney dysfunction	TLC, HPLC, 16S rRNA gene sequencing, DNA–DNA hybridization	[27]
*Gordonia iterans*	sputum	bacterial pneumonia	TLC, mycolic acid profile, 16S rRNA gene sequencing, DNA–DNA hybridization	[28]
*Gordonia aichiensis* (previously, *Rhodococcus aichiensis*)	sputum	pulmonary disease	mycolic acid profile, 16S rRNA sequencing	[29]
*Gordonia bronchialis*	sputum	cavitary pulmonary tuberculosis and/or bronchiectasis	mycolic acid profile, compound utilization patterns	[30]
*Gordonia sputi* (previously, *Rhodococcus sputi*)	sputum	pulmonary disease	mycolic acid profile, compound utilization patterns	[31]
*Gordonia jinhuaensis*	pharmaceutical wastewater	-	HPLC, 16S rRNA gene sequencing, compound utilization patterns	[32]
*Gordonia crocea*	drainage strips	wound infection after pacemaker implantation	MALDI TOF MS, 16S rRNA gene sequencing	[33]
*Gordonia hongkongensis*	(1) blood culture, (2) the peritoneal dialysis effluent	(1) continuous ambulatory peritoneal dialysis (CAPD)-related peritonitis, (2) bacteraemia	HPLC, 16S rRNA, gyrB, secA genes sequencing, DNA–DNA hybridization	[34]

Abbreviations: TLC—thin-layer chromatography; HPLC—high-performance liquid chromatography.

**Table 2:** *Gordonia* species, the type strains of which were initially isolated from environmental samples. Some strains of these species are human or veterinary pathogens.

Species	Disease	Reference to an Article with an Example of a Pathogenic Strain	Isolation Source of the Type Strain	Reference to an Article with Type Strain Isolation
*Gordonia amicalis*	cutaneous infection after a traumatic injury	[35]	soil contaminated with thiophenes	[10]
“*Gordonia jacobaea*”	prosthetic joint septic arthritis	[36]	soil	[37]
*Gordonia paraffinivorans*	cow mastitis	[38]	oil-producing well of Daqing oilfield	[39]
*Gordonia polyisoprenivorans*	catheter-related bacteremia	[40]	automobile tyre	[41]
*Gordonia rubripertincta*	lung infection	[42]	soil	[43]
*Gordonia sinesedis*	Lymphadenitis	[44]	soil	[45]
*Gordonia terrae*	acute cholecystitis	[46]	soil	[47]
*Gordonia westfalica*	mycetoma of the foot	[48]	foul water taken from the inside of a deteriorated automobile tyre	[14]

**Table 3:** Examples of matching the identification of *Gordonia* strains using the MALDI method and genetic methods.

Genus/Species Determined by the MALDI	Bruker Biotyper Version	Number of Identification Repetitions	Scores	The Result of Identification by an Additional Approach	The Approach Used for Additional Identification	Reference
*G. bronchialis*	ND	2	1.68, 2.08	*G. bronchialis*	16S rRNA	[76]
*G. bronchialis*	ND	1	1.83	*G. bronchialis*	*gyr*B	[65]
*G. rubripertincta*	3.0	1	1.764	*G. polyisoprenivorans*	16S rRNA, *gyr*B	[40]
*G. bronchialis*	ND	1	1.808	*G. bronchialis*	16S rRNA, *hsp*65	[77]
*G. sputi*	ND	2	1.65, 1.57	*G. sputi*	16S rRNA	[78]
*G. bronchialis*	ND	2	-	*G. bronchialis*	16S rRNA	[66] *
*G. sputi*	3.1	1	2.039	*G. sputi*	16S rRNA, *sec*A1	[79]
*G. sputi*		1	2.026	*G. sputi*	16S rRNA, *sec*A1	
*G. bronchialis*		1	1.743	*G. bronchialis*	16S rRNA, *sec*A1	
*Gordonia* sp.		1	1.550	*G. lacunae/G. terrae/*new species	16S rRNA, *sec*A1	
*G. rubripertincta*	ND	1	-	*G. bronchialis*	16S rRNA	[80]
*G. rubripertincta*	4.1	1	(for 27 samples) 1.723–2.319	*G. paraffinovorans*	WGS	[38]
*G. rubropertincta*	ND	1	1.702	*G. terrae*	16S rRNA	[56]

* The modified protocol was used because the standard one gave no result. ND—no data.

**Table 4:** Primers used for amplification of genes/fragments of 16S rRNA genes in the identification of *Gordonia*.

№	Primer Name	Primer Sequence	Species Identified	Reference
1	P8-27	5′-AGA GTT TGA TCC TGG CTC AG-3′	universal	[52,63,100]
	P1392-1372	5′-AAG GCC CGG GAA CGT ATT CAC-3′		
2	16S-F	5′-AGA GTT TGA TCC TGG CTC AG-3′	*G. bronchialis*	[76]
	16S-R	5′-ACG GCT ACC TTG TTA CGA CTT-3′		
3	fD1	5′-CCG AAT TCG TCG ACA ACA GAG TTT GAT CCT GGC TCA G-3′	*G. polyisoprenivorans*	[54]
	rD1	5′-CCC GGG ATC CAA GCT TAA GGA GGT GAT CCA GCC-3′		
4	27FLP	5′-AGA GTT TGA TCM TGG CTC AG-3′	*G. terrae*	[55]
	1492RPL	5′-GGT TAC CTT GTT ACG ACT T-3′		
5	-	5′-TGG AGA GTT TGA TCC TGG CTC AG-3′	*G. bronchialis*	[101]
	-	5′-TAC CGC GGC TGC TGG CAC-3′		
6	BACT	5′-CAG GCC TAA CAC ATG CAA GTC-3′	*G. sputi*	[58]
	UNI	5′-GAC GGG CGG TGT GTA CAA-3′		
7	G268F	5′-CGA CCT GAG AGG GTG ATC G-3′	*G. sputi*, *G. bronchialis*, *G. terrae/lacumae*	[79,102]
	G1096R	5′-ATA ACC CGC TGG CAA TAC AG-3′		
8	8FLP	5′-AGA GTT TGA TCC TGG CTC AG-3′	*G. terrae*, *G. sputi*	[103]
	1492RPL	5′-GGT TAC CTT GTT ACG ACT T-3′		
9	5F	5′-TTG GAG AGT TTG ATC CTG GCT C-3′	*G. terrae*, *G. otitidis*, *G. bronchialis*	[52]
	1194R	5′-ACG TCA TCC CCA CCT TCC TC-3′		
10	27FI	5′-AGA GTT TGA TCC TGG CTC AG-3′	*G. sputi*	[104,105]
	1494Rc	5′-TAC GGC TAC CTT GTT ACG AC-3′		
11	fD1	5′-CCG AAT TCG TCG ACA ACA GAG TTT GAT CCT GGC TCA G-3′	*G. bronchialis*	[106,107]
	rP2	5′-CCC GGG ATC CAA GCT TAC GGC TAC CTT GTT ACG ACT T-3′		
12	27F	5′-GAG TTT GAT CCT GGC TCA G-3′	*G. terrae*	[56]
	1492R	5′-AAG GAG GTG ATC CAG CCG CA-3′		
13	5F	5′-TGG AGA GTT TGA TCC TGG CTA G-3′	*G. sputi*, *G. otitidis*, *G. bronchialis*	[71]
	1193R	5′-ACG TCA TCC CCG CTT CCT T-3′		
14	w001	5′-AGA GTT TGA TCM TGG CTC-3′	*G. bronchialis*	[80,108]
	w002	5′-GNT ACC TTG TTA CGA CTT-3′		

**Table 5:** Sequences of primers used for amplification of *gyr*B genes/fragments in *Gordonia* strains.

Primer Name	Primer Sequence	Species	Reference
GYRB1	5′-ATG CAN CAR YTN CAY GCN GGN-3′	universal	[118]
GYRB2	5′-SAY GAT CTT GTK RTA SCG MAA YTT-3′		
UP-1	5′-GAA GTC ATC ATG ACC GTT CTG CAY GCN GGN GGN AAR TTY GA-3′	universal	[120]
UP-2	5′-AGC AGG GTA CGG ATG TGC GAG CCR TCN ACR TCN GCR TCN GTC AT-3′		
UP1F	5′-GAG GTC GTC ATG ACC CAG CTG CAY GCN GGN GGN AAR TTY GA-3′	*G. otitidis*	[50]
UP2r-modi	5′-AGC AGC GTC GAG ATG TGC TGG CCR TCN ACR TCN GCR TCN GTC A-3′		

**Table 6:** Sequences of primers used for amplification of *sec*A genes/fragments in *Gordonia* strains.

Primer Name	Primer Sequence	Reference
SecA1-f	5′-GTA AAA CGA CGG CCA GGA CAG YGA GTG GAT GGG YCG SGT GCA CCG-3′	[114]
SecA1-r	5′-CAG GAA ACA GCT ATG ACG CGG ACG ATG TAG TCC TTG TC-3′	

## Data Availability

Not applicable.
